# Supplementary figures and images for: A point mutation in the nucleotide exchange factor eIF2B constitutively activates the integrated stress response by allosteric modulation
Source: eLife. 2022 Apr 13;11:e76171. doi: 10.7554/eLife.76171 (PMC9132573; doi:10.7554/eLife.76171)

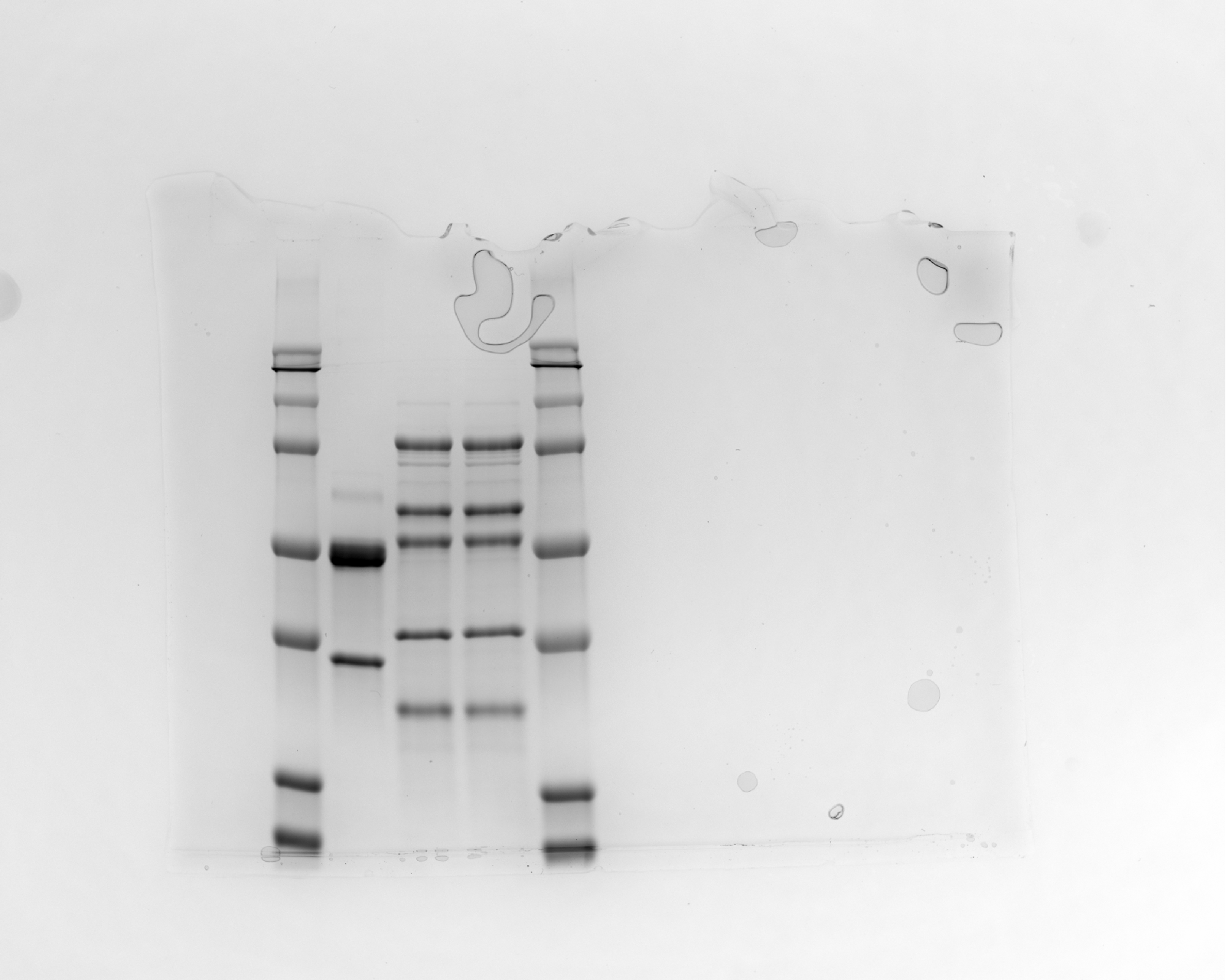

Supplement: Figure 1—figure supplement 1—source data 1. [file elife-76171-fig1-figsupp1-data1.zip › Fig_1_sup_1_source_data_1.tif]

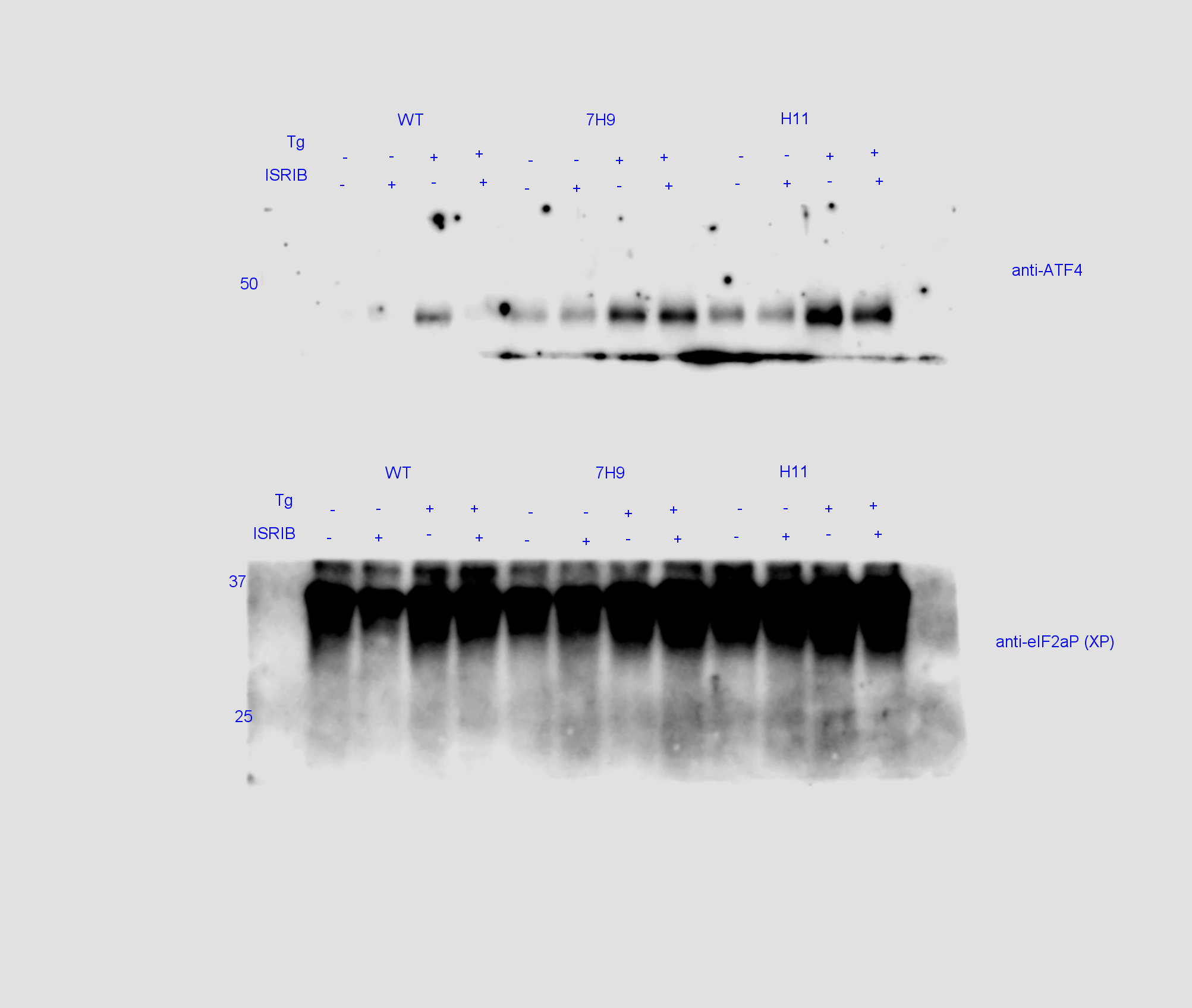

Supplement: Figure 6—source data 2. — Tg: thapsigargin; 7H9: homozygous βH160D clone (βH160D #1); H11: hemizygous βH160D clone (βH160D #2). [file elife-76171-fig6-data2.zip › Fig_6_source_data_2_blot_ATF4.tif]

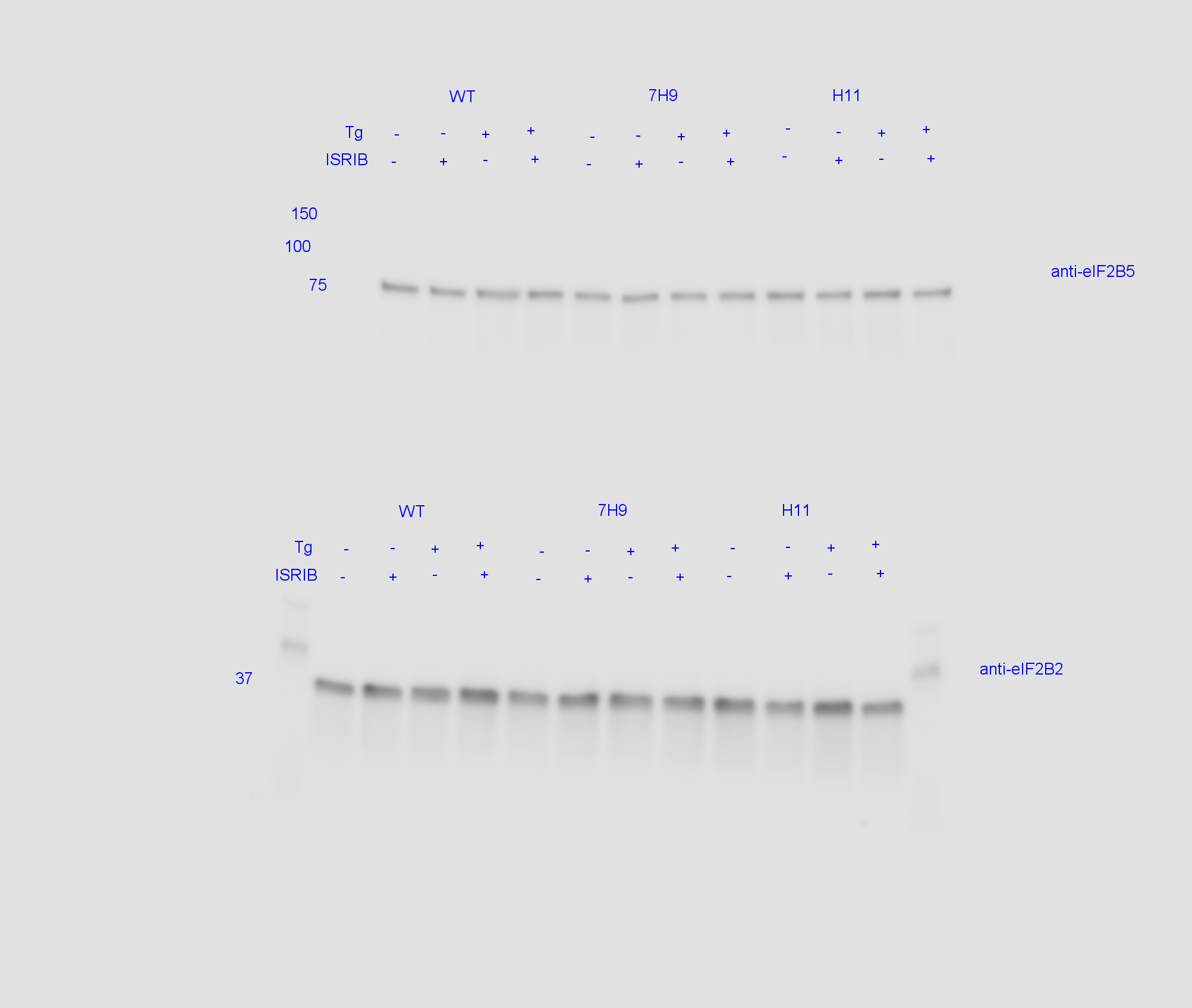

Supplement: Figure 6—source data 2. — Tg: thapsigargin; 7H9: homozygous βH160D clone (βH160D #1); H11: hemizygous βH160D clone (βH160D #2). [file elife-76171-fig6-data2.zip › Fig_6_source_data_2_blot_eIF2B2.tif]

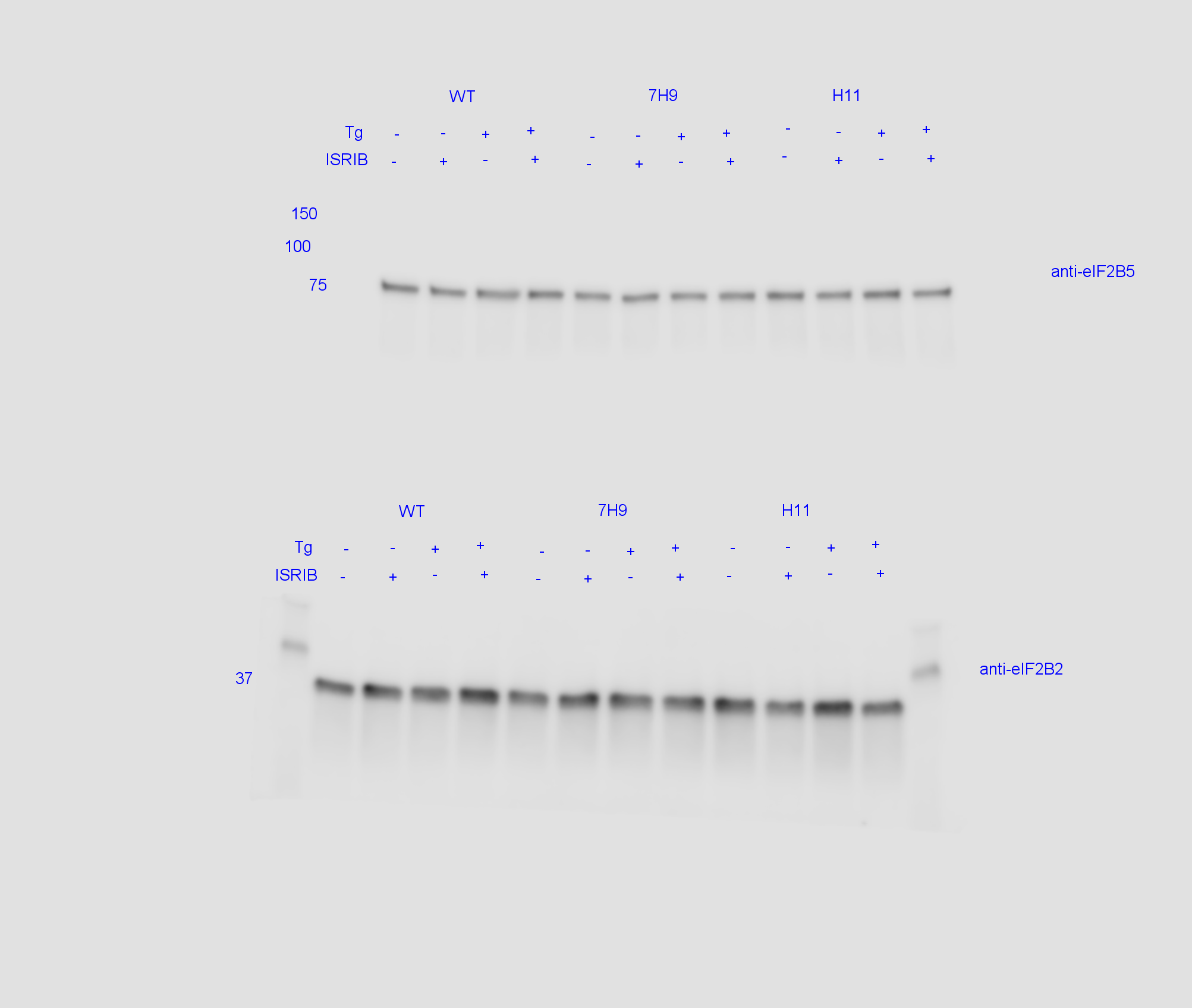

Supplement: Figure 6—source data 2. — Tg: thapsigargin; 7H9: homozygous βH160D clone (βH160D #1); H11: hemizygous βH160D clone (βH160D #2). [file elife-76171-fig6-data2.zip › Fig_6_source_data_2_blot_eIF2B5.tif]

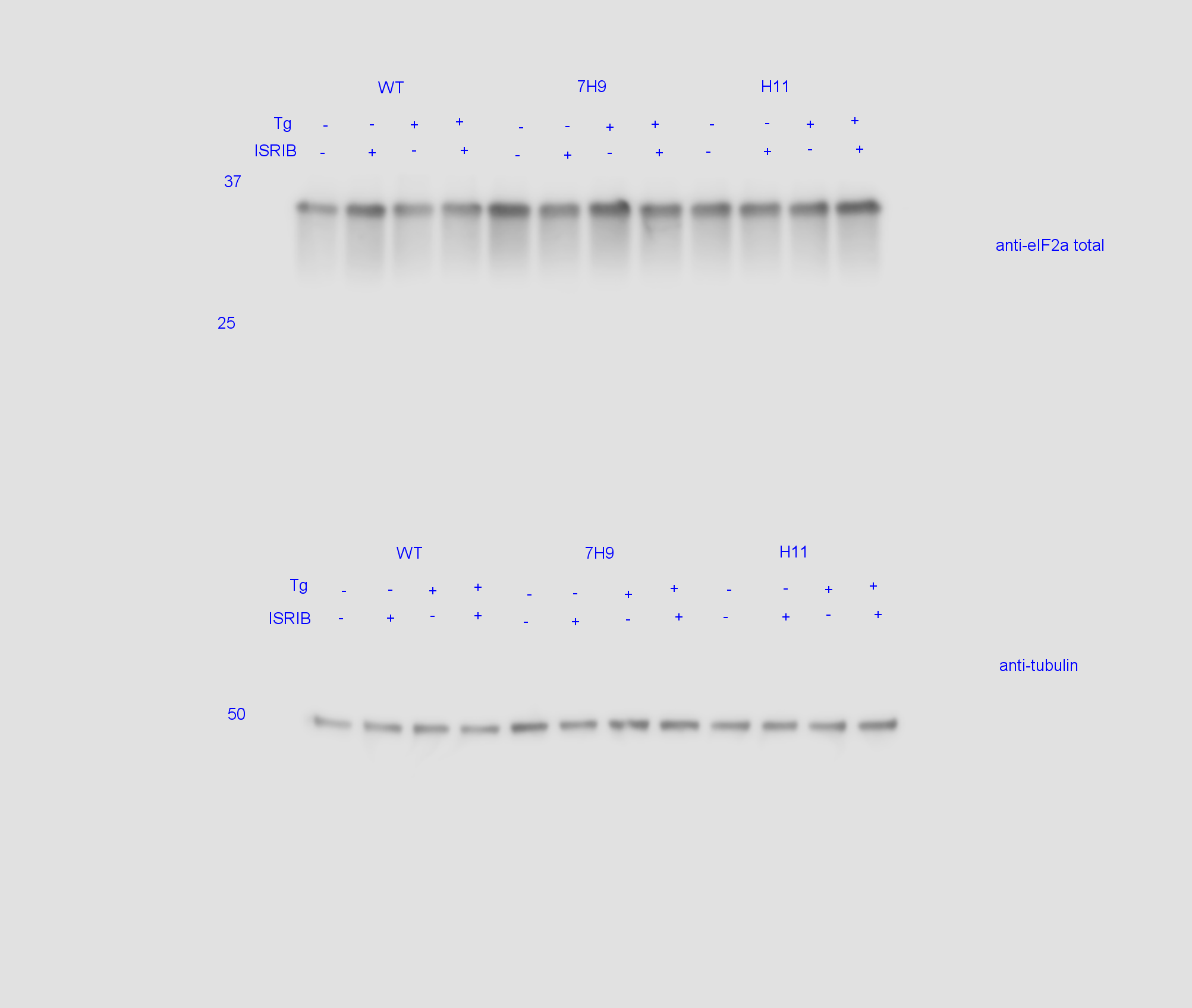

Supplement: Figure 6—source data 2. — Tg: thapsigargin; 7H9: homozygous βH160D clone (βH160D #1); H11: hemizygous βH160D clone (βH160D #2). [file elife-76171-fig6-data2.zip › Fig_6_source_data_2_blot_tubulin.tif]

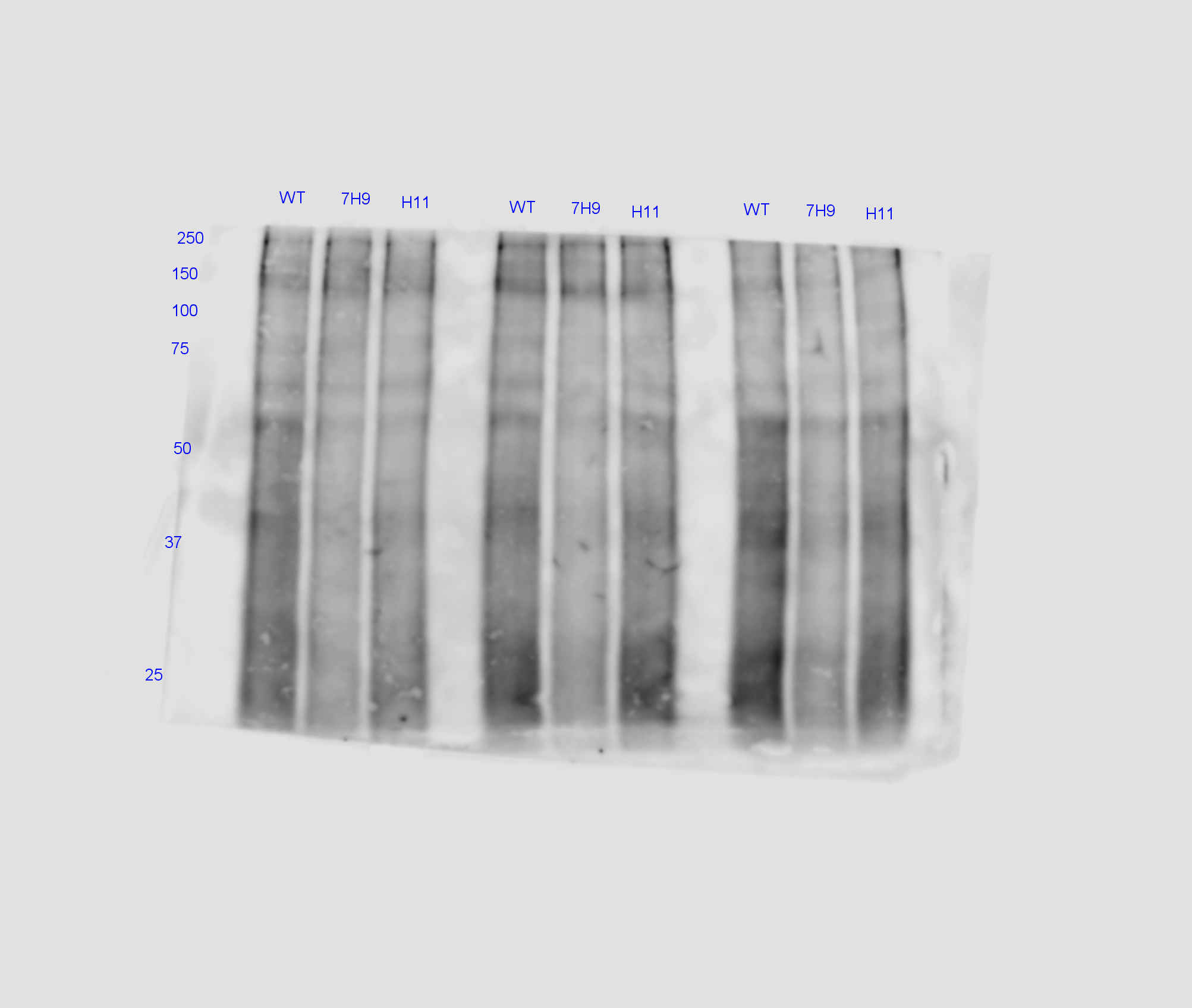

Supplement: Figure 6—figure supplement 2—source data 2. — 7H9 = homozygous βH160D clone (βH160D #1), H11 = hemizygous βH160D clone (βH160D #2). [file elife-76171-fig6-figsupp2-data2.zip › Fig_6_sup_2_source_data_2_puro_rep1.tif]

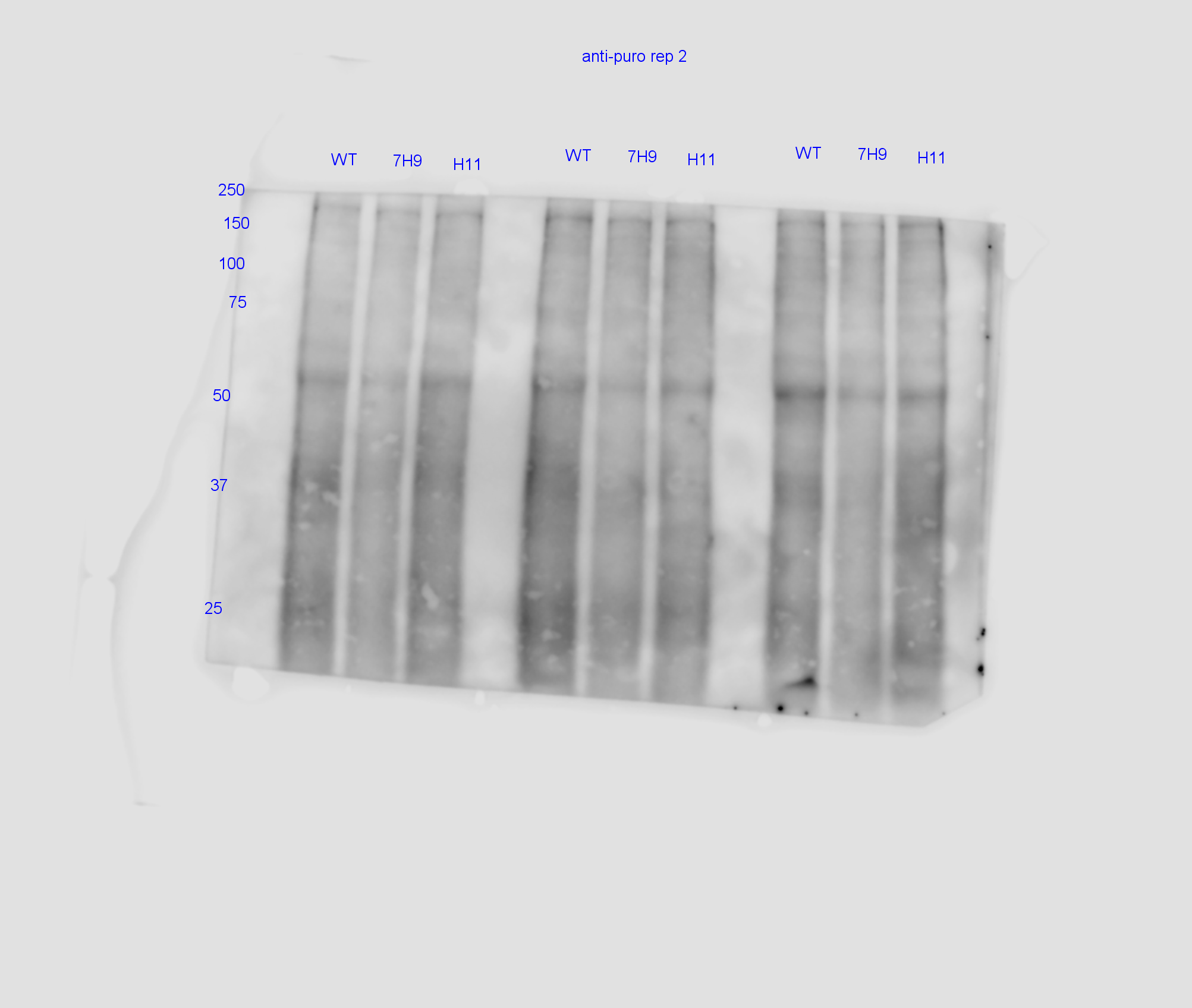

Supplement: Figure 6—figure supplement 2—source data 2. — 7H9 = homozygous βH160D clone (βH160D #1), H11 = hemizygous βH160D clone (βH160D #2). [file elife-76171-fig6-figsupp2-data2.zip › Fig_6_sup_2_source_data_2_puro_rep2.tif]

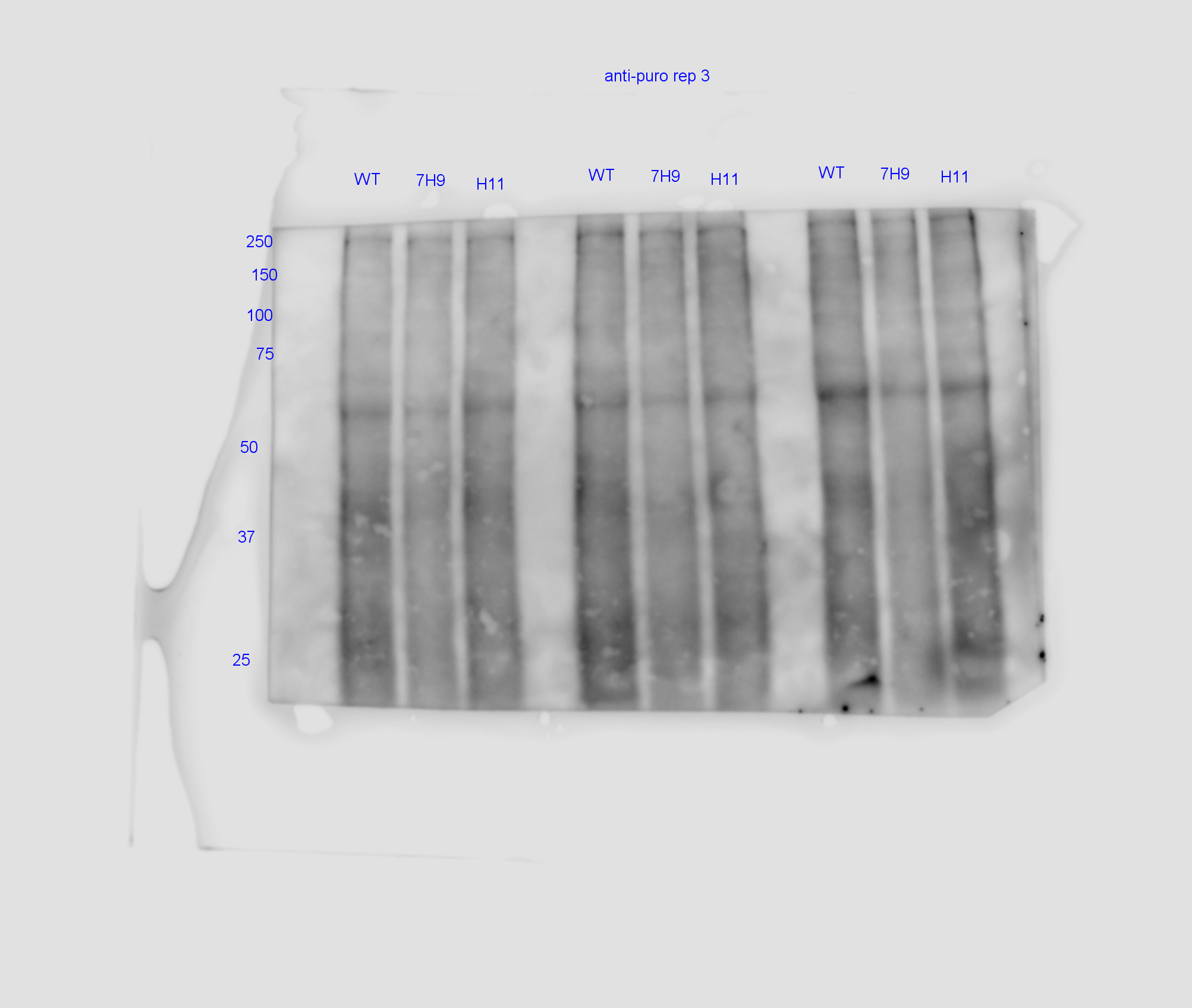

Supplement: Figure 6—figure supplement 2—source data 2. — 7H9 = homozygous βH160D clone (βH160D #1), H11 = hemizygous βH160D clone (βH160D #2). [file elife-76171-fig6-figsupp2-data2.zip › Fig_6_sup_2_source_data_2_puro_rep3.tif]

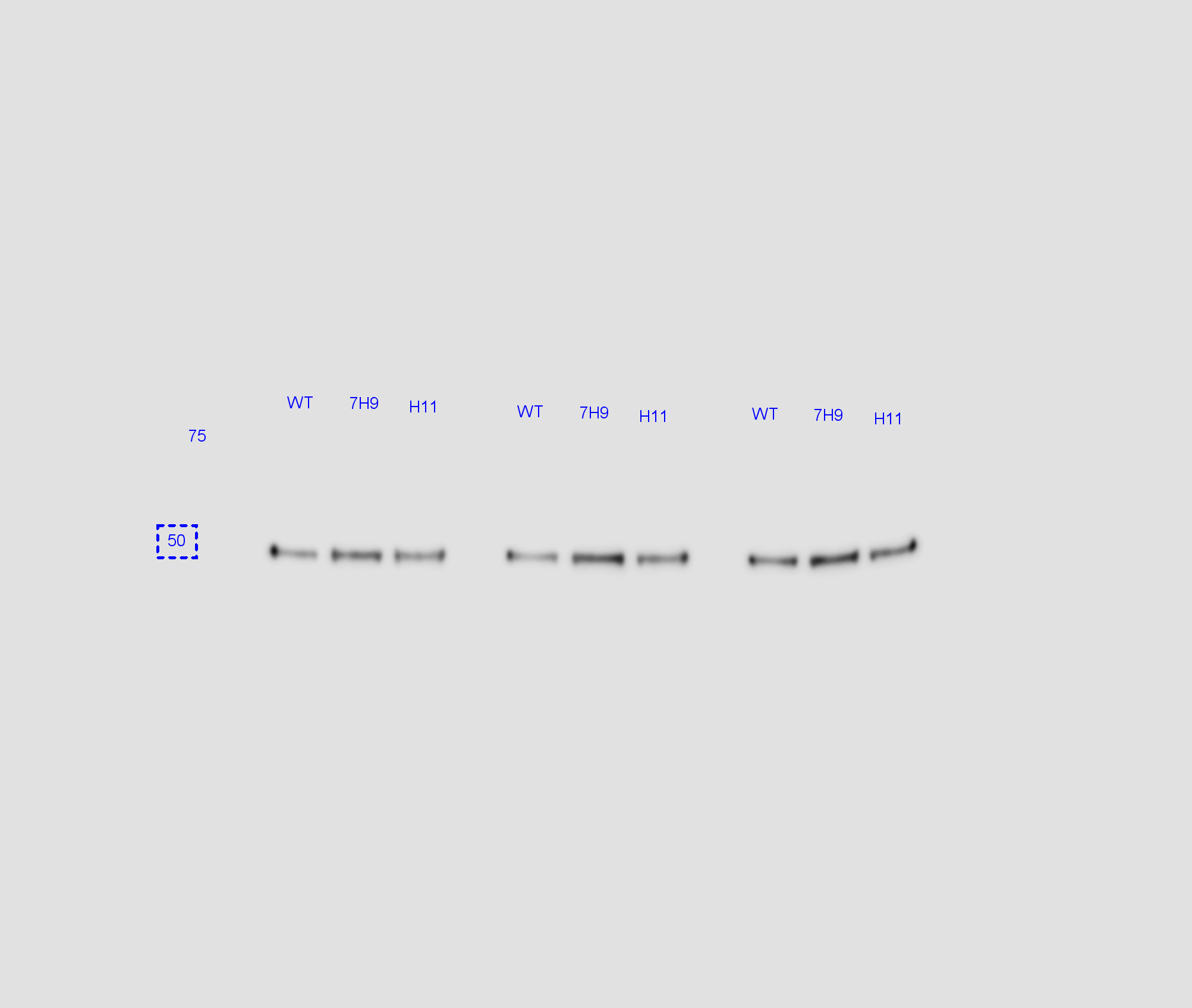

Supplement: Figure 6—figure supplement 2—source data 2. — 7H9 = homozygous βH160D clone (βH160D #1), H11 = hemizygous βH160D clone (βH160D #2). [file elife-76171-fig6-figsupp2-data2.zip › Fig_6_sup_2_source_data_2_tub_rep1.tif]

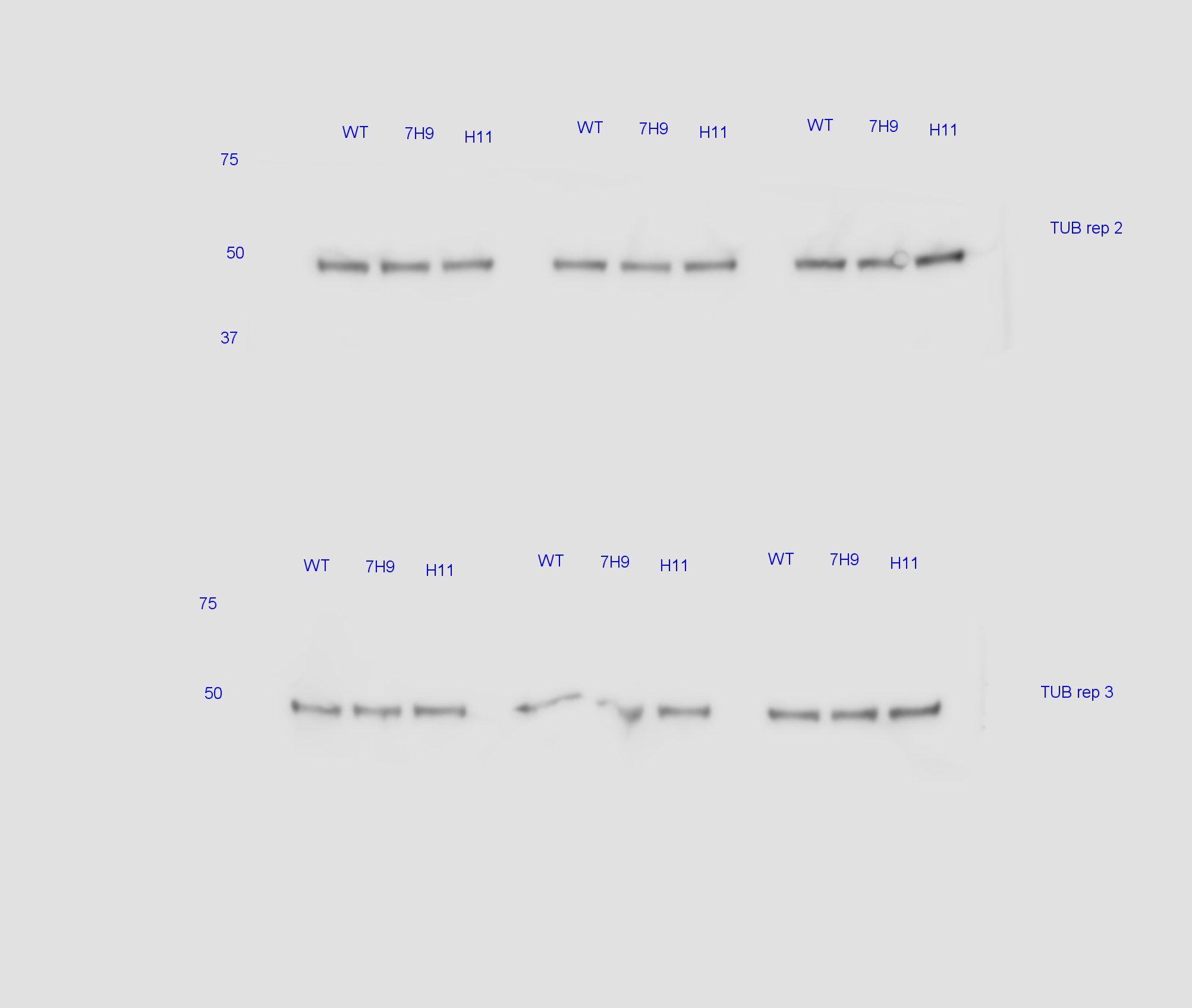

Supplement: Figure 6—figure supplement 2—source data 2. — 7H9 = homozygous βH160D clone (βH160D #1), H11 = hemizygous βH160D clone (βH160D #2). [file elife-76171-fig6-figsupp2-data2.zip › Fig_6_sup_2_source_data_2_tub_rep2andrep3.tif]

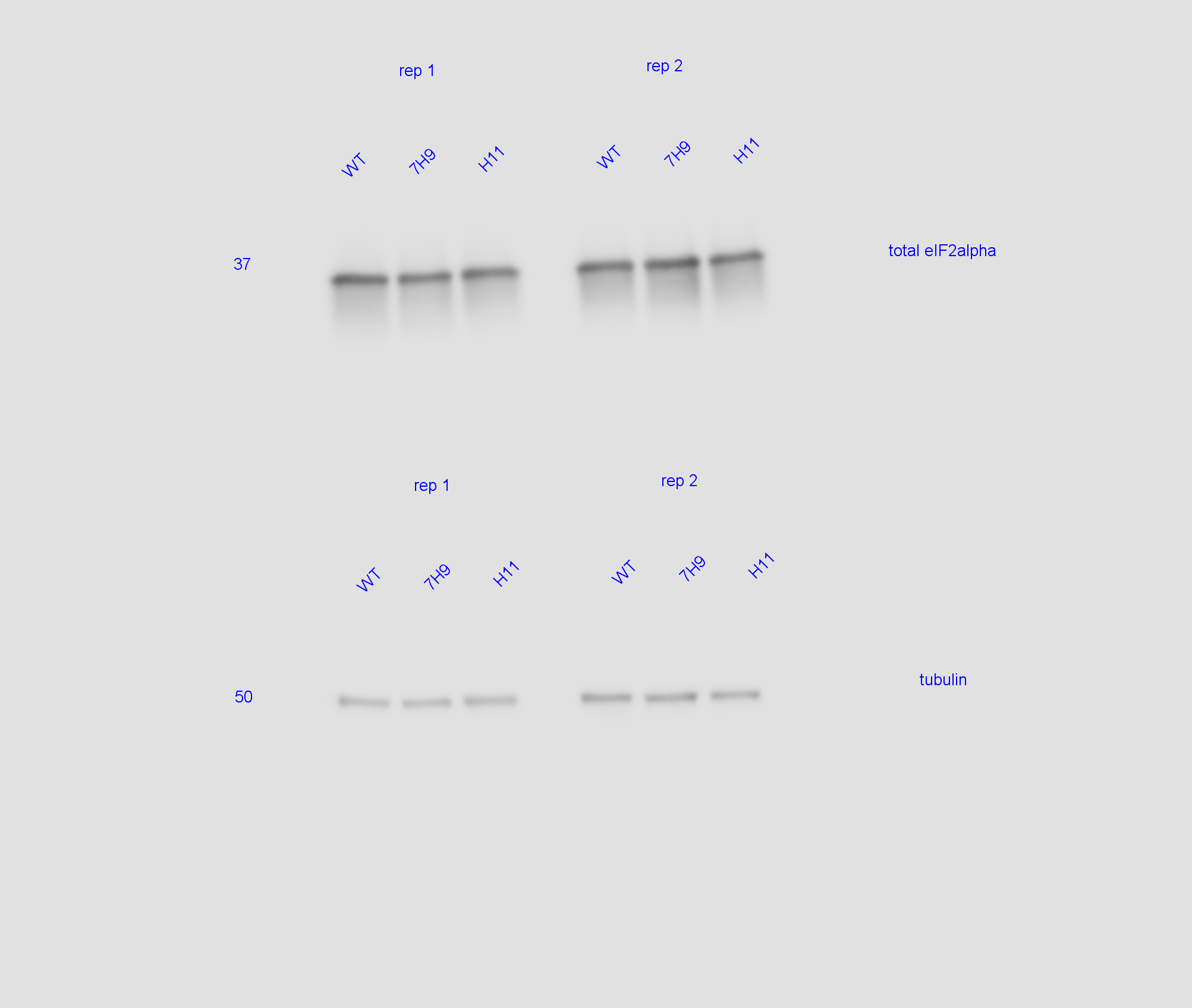

Supplement: Figure 6—figure supplement 3—source data 2. — 7H9: homozygous βH160D clone (βH160D #1); H11: hemizygous βH160D clone (βH160D #2). [file elife-76171-fig6-figsupp3-data2.zip › Fig_6_sup_3_source_data_2_eIF2a.tif]

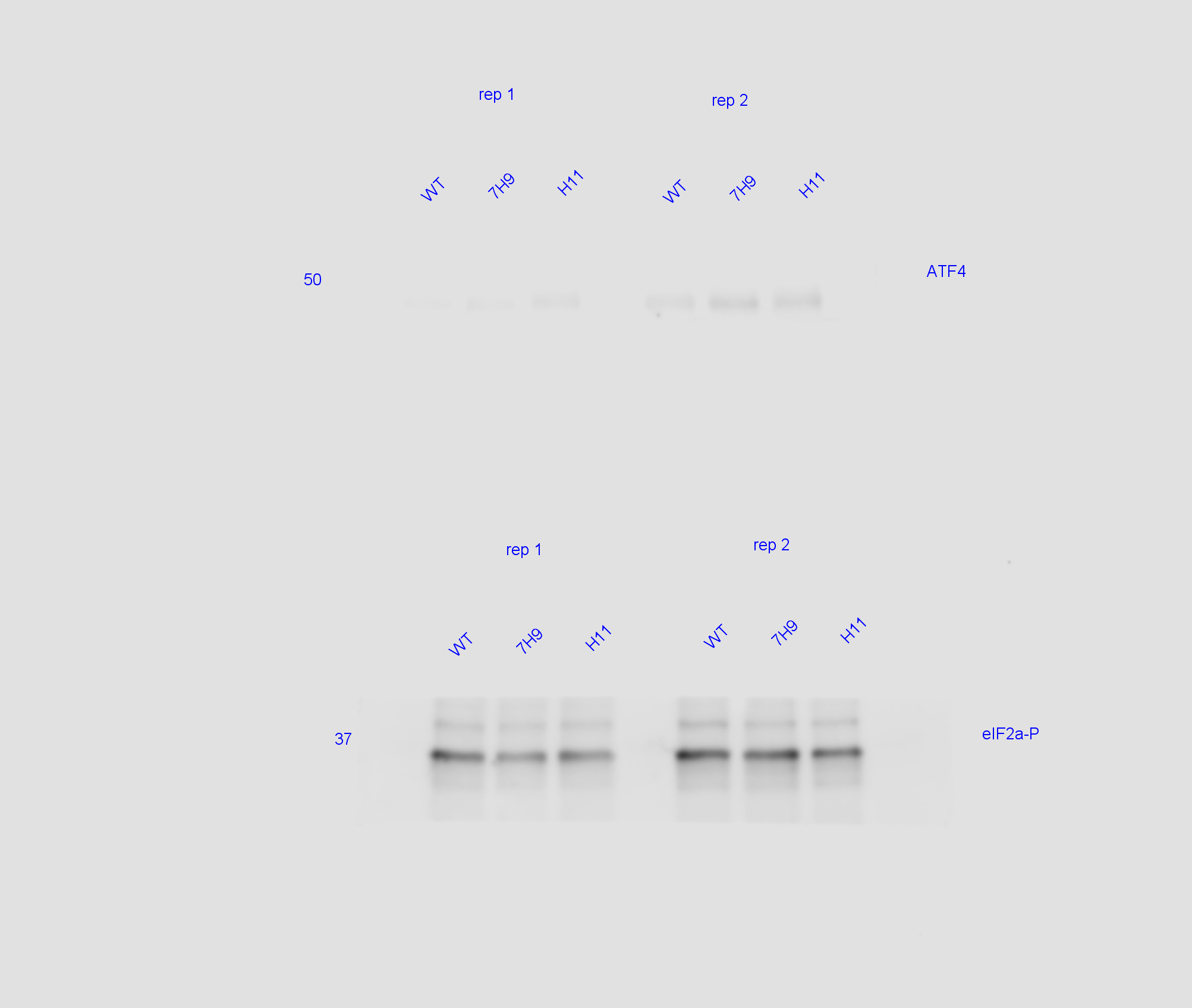

Supplement: Figure 6—figure supplement 3—source data 2. — 7H9: homozygous βH160D clone (βH160D #1); H11: hemizygous βH160D clone (βH160D #2). [file elife-76171-fig6-figsupp3-data2.zip › Fig_6_sup_3_source_data_2_eIF2aP.tif]

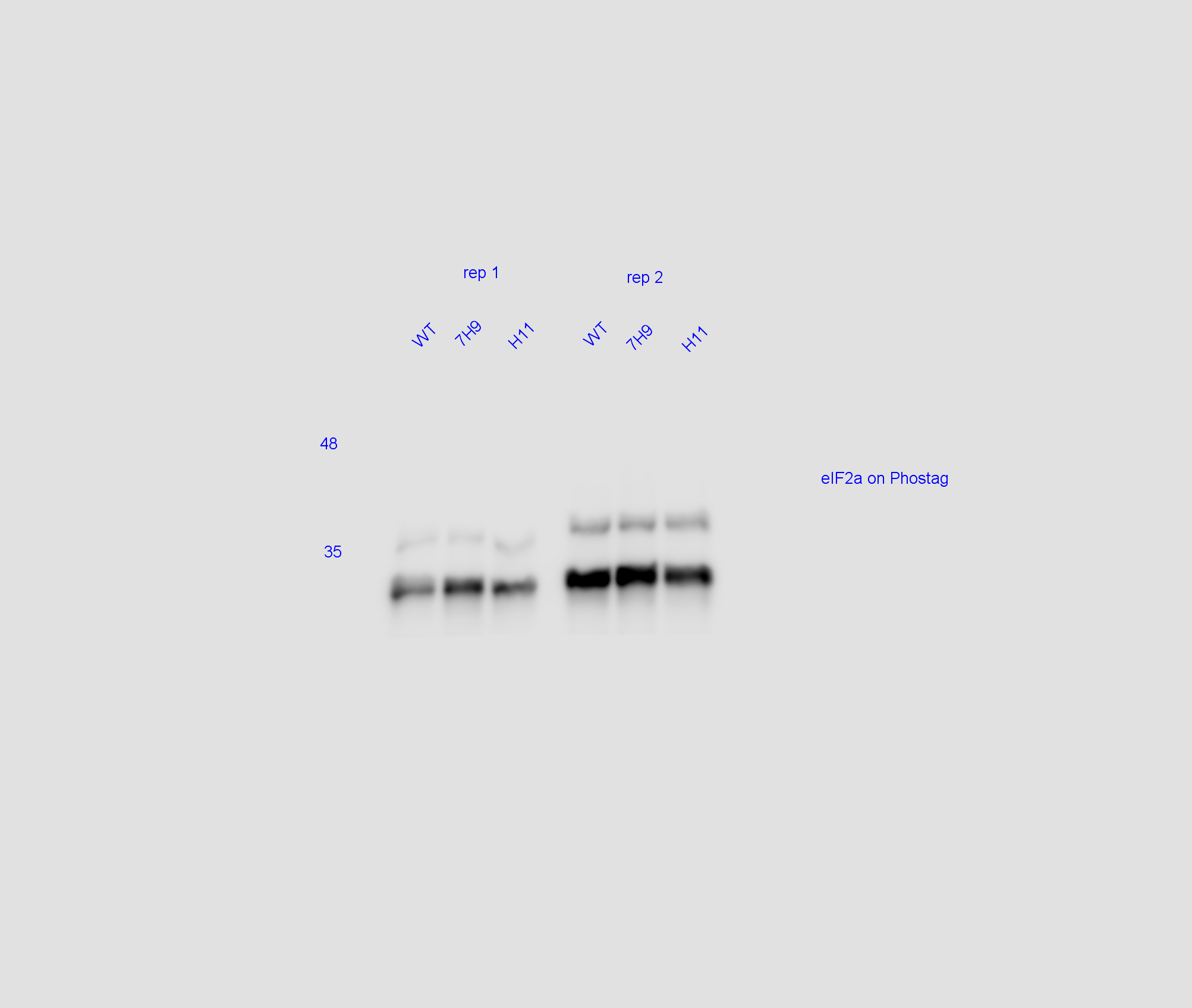

Supplement: Figure 6—figure supplement 3—source data 2. — 7H9: homozygous βH160D clone (βH160D #1); H11: hemizygous βH160D clone (βH160D #2). [file elife-76171-fig6-figsupp3-data2.zip › Fig_6_sup_3_source_data_2_Phostag_eIF2_rep1-exposure.tif]

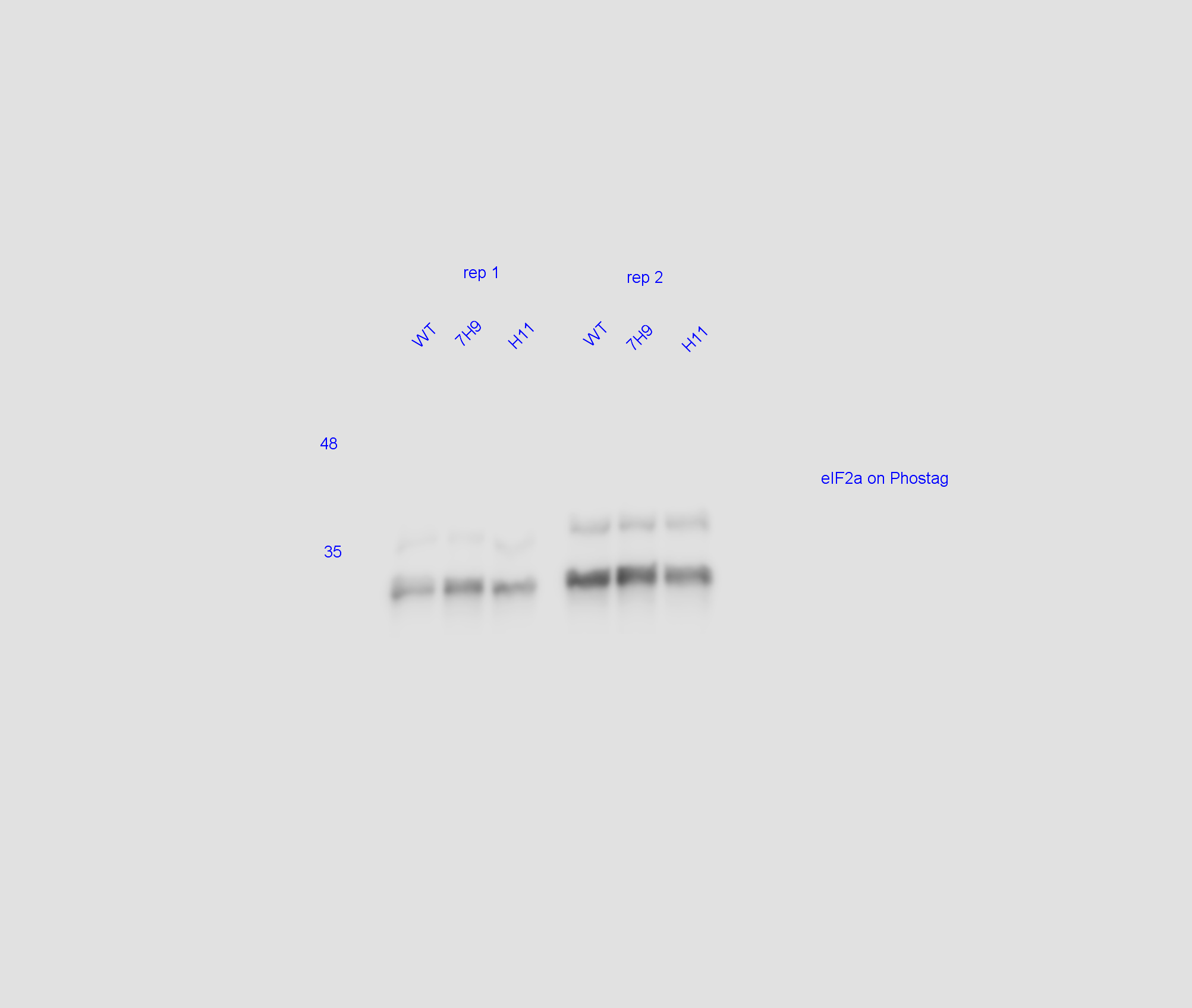

Supplement: Figure 6—figure supplement 3—source data 2. — 7H9: homozygous βH160D clone (βH160D #1); H11: hemizygous βH160D clone (βH160D #2). [file elife-76171-fig6-figsupp3-data2.zip › Fig_6_sup_3_source_data_2_Phostag_eIF2_rep2-exposure.tif]

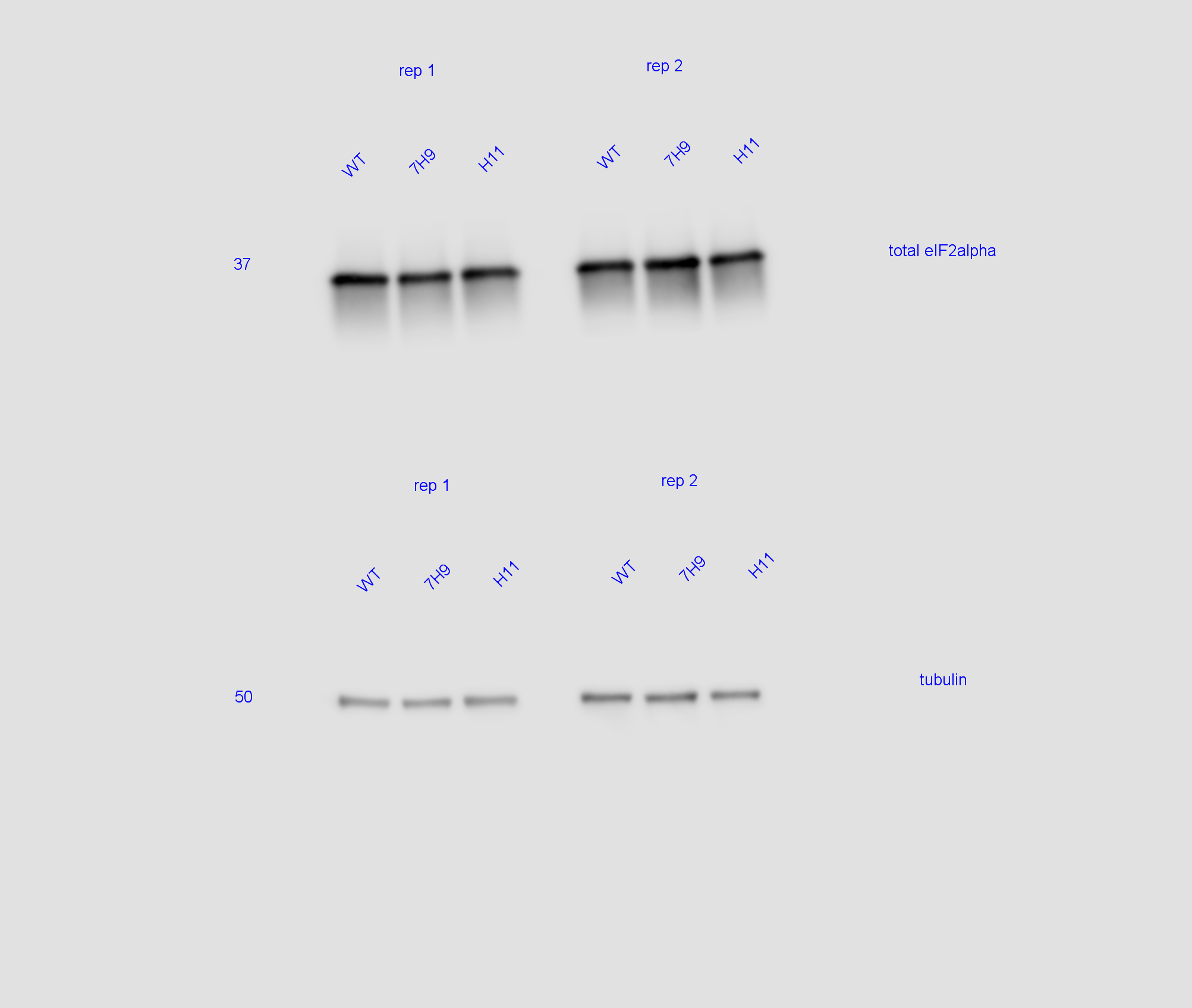

Supplement: Figure 6—figure supplement 3—source data 2. — 7H9: homozygous βH160D clone (βH160D #1); H11: hemizygous βH160D clone (βH160D #2). [file elife-76171-fig6-figsupp3-data2.zip › Fig_6_sup_3_source_data_2_tub.tif]
